# The Paradoxical Effect of Creatine Monohydrate on Muscle Damage Markers: A Systematic Review and Meta-Analysis

**DOI:** 10.1007/s40279-022-01640-z

**Published:** 2022-02-26

**Authors:** Kenji Doma, Akhilesh Kumar Ramachandran, Daniel Boullosa, Jonathan Connor

**Affiliations:** 1 James Cook Drive, Rehabilitation Sciences Building, College of Healthcare Sciences, Sports and Exercise Science, James Cook University, Douglas, QLD QLD481 Australia; 2 Sports Dynamix Private Limited, Chennai, India; 3 Federal University of Mato Grosso, Mato Grosso, Brazil

## Abstract

**Background:**

Several studies have examined the effect of creatine monohydrate (CrM) on indirect muscle damage markers and muscle performance, although pooled data from several studies indicate that the benefits of CrM on recovery dynamics are limited.

**Objective:**

This systematic review and meta-analysis determined whether the ergogenic effects of CrM ameliorated markers of muscle damage and performance following muscle-damaging exercises.

**Methods:**

In total, 23 studies were included, consisting of 240 participants in the CrM group (age 23.9 ± 10.4 years, height 178 ± 5 cm, body mass 76.9 ± 7.6 kg, females 10.4%) and 229 participants in the placebo group (age 23.7 ± 8.5 years, height 177 ± 5 cm, body mass 77.0 ± 6.6 kg, females 10.0%). These studies were rated as fair to excellent following the PEDro scale. The outcome measures were compared between the CrM and placebo groups at 24–36 h and 48–90 h following muscle-damaging exercises, using standardised mean differences (SMDs) and associated *p*-values via forest plots. Furthermore, sub-group analyses were conducted by separating studies into those that examined the effects of CrM as an acute training response (i.e., after one muscle-damaging exercise bout) and those that examined the chronic training response (i.e., examining the acute response after the last training session following several weeks of training).

**Results:**

According to the meta-analysis, the CrM group exhibited significantly lower indirect muscle damage markers (i.e., creatine kinase, lactate dehydrogenase, and/or myoglobin) at 48–90 h post-exercise for the acute training response (SMD − 1.09; *p* = 0.03). However, indirect muscle damage markers were significantly greater in the CrM group at 24 h post-exercise (SMD 0.95; *p* = 0.04) for the chronic training response. Although not significant, a large difference in indirect muscle damage markers was also found at 48 h post-exercise (SMD 1.24) for the chronic training response. The CrM group also showed lower inflammation for the acute training response at 24–36 h post-exercise and 48–90 h post-exercise with a large effect size (SMD − 1.38 ≤ *d* ≤  − 1.79). Similarly, the oxidative stress markers were lower for the acute training response in the CrM group at 24–36 h post-exercise and 90 h post-exercise, with a large effect size (SMD − 1.37 and − 1.36, respectively). For delayed-onset muscle soreness (DOMS), the measures were lower for the CrM group at 24 h post-exercise with a moderate effect size (SMD − 0.66) as an acute training response. However, the inter-group differences for inflammation, oxidative stress, and DOMS were not statistically significant (*p* > 0.05).

**Conclusion:**

Overall, our meta-analysis demonstrated a paradoxical effect of CrM supplementation post-exercise, where CrM appears to minimise exercise-induced muscle damage as an acute training response, although this trend is reversed as a chronic training response. Thus, CrM may be effective in reducing the level of exercise-induced muscle damage following a single bout of strenuous exercises, although training-induced stress could be exacerbated following long-term supplementation of CrM. Although long-term usage of CrM is known to enhance training adaptations, whether the increased level of exercise-induced muscle damage as a chronic training response may provide potential mechanisms to enhance chronic training adaptations with CrM supplementation remains to be confirmed.

**Supplementary Information:**

The online version contains supplementary material available at 10.1007/s40279-022-01640-z.

## Key Points

**Table:** 

Creatine monohydrate reduced the level of exercise-induced muscle damage as an acute training response, although this trend was reversed as a chronic training response.
Creatine monohydrate may be an ergogenic supplement to accelerate recovery following a single bout of strenuous exercise.
The increased level of exercise-induced muscle damage after several weeks of training and creatine monohydrate supplementation may suggest a possibly greater tolerance of training stresses, given that long-term creatine monohydrate supplementation is known to enhance training adaptation.

## Introduction

Exercise-induced muscle damage (EIMD) is a common phenomenon following muscular contractions, particularly with unfamiliar activities, eccentric contractions, or those under heavy loads [1]. The common symptoms of EIMD include increased serum muscle proteins (e.g., creatine kinase [CK]), inflammatory markers (e.g., interleukin [IL]-6), oxidative stress (e.g., hydrogen peroxide), delayed onset of muscle soreness (DOMS), and prolonged impairment in functional performance [2–4]. Furthermore, EIMD impairs running [5–7], cycling power output [8], and sprint and agility performances [9, 10], which may induce sub-optimal training adaptation if symptoms of EIMD are extended [11]. However, effective dietary supplements may minimise the negative effects of EIMD without compromising long-term adaptations [12], thus reducing the need for resting days and reduced workload to favour appropriate recovery between training bouts.

Several commercially available oral supplements are known to minimise the level of EIMD, including extracts derived from fruits [13], tea leaf [14], and root plants [15], which contain a high amount of antioxidant and anti-inflammatory properties. Although these supplements may aid in recovery following strenuous exercise and enhance preparedness for athletes between training bouts, the benefits in terms of enhanced chronic training adaptations are not well documented. On the other hand, creatine monohydrate (CrM) has long been used as an oral supplement to enhance muscular strength [16] and hypertrophic adaptations [17] when ingested during resistance training periods. A number of mechanisms have been proposed to explain the benefit that CrM has on resistance training-induced adaptations, including larger lean body mass [18], increased protein expression and synthesis [19], changes in myogenic transcription factors [20], and elevated mitotic activity of satellite cells [21]. Above all, the most likely benefit appears to be due to improved performance during resistance training sessions by increasing intra-muscular phosphocreatine stores, thereby allowing a greater work capacity and thus training stimuli for enhanced chronic training adaptation [22, 23]. Recent evidence also suggests the potential for CrM supplementation to attenuate muscle damage markers as an acute response to exercise [24]. The mechanical disruption of sarcomeres is considered as the initial event of muscular injury [1]. This primary muscle damage is followed by inflammatory and oxidative stress responses, referred to as the secondary muscle damage response, which increases vascular permeability, oedema, and leukocyte infiltration, resulting in further muscle damage, thus compromising the recovery of muscle structure and function [1]. Interestingly, CrM has been reported to acutely reduce biomarkers of oxidative stress following strenuous exercise in rats [25], with in vitro data indicating that CrM exhibits anti-inflammatory effects in endothelial cells [26]. Although these findings may not be directly translatable to humans, they suggest that CrM possesses both anti-inflammatory and antioxidant properties, which would attenuate the secondary muscle damage response.

Several studies have reported reductions in CK, DOMS, inflammation, and oxidative stress, with a concomitant increase in muscle strength following a single bout of strenuous exercise in individuals supplemented with CrM compared with those receiving placebo [27–29] for 24–48 h post-exercise. Ingestion of CrM may enhance recovery following strenuous exercise that causes EIMD. In fact, Claudino et al. [30] reported that the level of decrement in lower-body power output was reduced with the ingestion of CrM when compared with placebo during the pre-season in elite soccer players, thus demonstrating the potential benefit of CrM to minimise non-functional overreaching. However, when examining the acute responses of the last training session following several weeks of resistance training, studies also reported greater levels of EIMD markers for the CrM group when compared with the placebo group, up to 48 h post-exercise, despite greater increases in muscle strength in the CrM group [31–33]. Therefore, it would appear that CrM exhibits a paradoxical effect on the EIMD response, whereby this supplement minimises the level of EIMD following a single bout of unfamiliar exercises, although this is reversed if supplemented for several weeks as part of a training programme.

It has been postulated that the increased work capacity typically observed with CrM supplementation may augment the rate of progression of training intensity or volume, resulting in superior long-term training adaptation [33]. However, this accelerated progression in training variables may also increase the level of acute physiological stresses [32]. In addition, the ergogenic effects of CrM for recovery vary between 1 and 3 days after strenuous exercises between studies and type of outcome measures, demonstrating the importance of capturing post-exercise stress responses over several days. Furthermore, other studies have also shown no benefit of CrM supplementation on outcome measures associated with EIMD [34–38]. Thus, the effect of CrM on EIMD markers appears conflicting to date, possibly because of distinct methodologies between previous studies, such as the supplementation methods of CrM, training background of participants, the type of muscle-damaging protocol, and EIMD outcome measures.

Therefore, a systematic exploration that addresses the methodological discrepancies in these previous studies may clarify the acute and chronic effectiveness of CrM supplementation to minimise EIMD symptoms in different settings. Of note, a systematic review and meta-analysis was conducted recently on the ergogenic effects of CrM on muscle damage markers [24], with findings indicating that CrM may reduce CK at 48 h post-exercise, although inconclusive results were reported for muscle strength, muscle soreness, and joint range of motion. Although these findings provide important evidence on the effects of CrM based on pooled data from several studies, the chronic effects of training and CrM supplementation on muscle damage markers have not yet been examined. Furthermore, inflammatory and oxidative stress markers during periods of EIMD are indicative of the secondary muscle damage response [1], although these biomarkers were not assessed in the systematic review by Northeast and Clifford [24]. Expanding on these outcome measures may demonstrate further impact of CrM as an ergogenic aid and shed light on the potential mechanisms underpinning greater training adaptation. Therefore, the purpose of the current systematic review and meta-analysis was twofold: to investigate the effect of CrM supplementation on various biomarkers (indirect muscle damage, inflammation, and oxidative stress) and muscular strength measures following a single bout of strenuous activities and to examine these outcome measures following the last bout of several weeks of training.

## Methodology

This systematic review has been registered with PROSPERO (registration number: CRD42020207421) and was conducted following the PRISMA (Preferred Reporting Items for Systematic Reviews and Meta-Analyses) guidelines [39]. The PICO (population, intervention/exposure, comparison and outcome) approach was used to construct the inclusion criteria, with the following inclusion and exclusion criteria:

- Population: healthy male and female humans.
- Intervention: ingestion of oral CrM supplements.
- Exposure: exercises employed to cause EIMD, such as isokinetic eccentric contractions, resistance training, plyometrics, and running.
- Comparison: the outcome measures were compared between the CrM and placebo groups at 24–36 and 48–90 h after the muscle-damaging exercises.
- Outcome: the outcome measures included blood biomarkers of indirect muscle damage (i.e., CK, myoglobin and lactate dehydrogenase [LDH]) and subjective measures of muscle soreness (i.e., visual analogue scale) and muscular performance (i.e., isometric or isokinetic torque, vertical jump and maximum strength).

The exclusion criteria were as follows: (1) studies conducted in animals; (2) studies where CrM supplements were used to induce chronic adaptations, such as assessment of strength development after 6 weeks of resistance training with CrM supplementation without measurement of indirect muscle damage, muscle soreness, and acute responses to muscular performance; (3) studies with outcome measures reported immediately after (< 24 h) or > 90 h after the muscle-damaging exercises; (4) studies published in non-English languages; (5) study results published as conference proceedings, reviews, and case reports.

### Search Strategy

A literature search was performed from 27 August 2021 using the PubMed, Scopus, SPORTDiscus, CINAHL, and Web of Science databases. Four strings of medical subject heading terms were employed for the PubMed search: (1) adult or young adult; (2) supplements (dietary supplements); (3) indirect muscle damage markers (creatine kinase; muscle, skeletal; l-lactate dehydrogenase; pain/drug therapy; pain/aetiology; muscle fatigue/drug effects; muscle fatigue/physiology; myalgia/drug therapy; myalgia/prevention and control); and (4) exercise (exercise test; exercise tolerance/physiology; physical endurance/physiology; physical exertion/physiology; physical endurance/drug effects; exercise; resistance training; muscle contraction; running/physiology). A free-text search was conducted for CINAHL, Scopus, SPORTDiscus, and Web of Science with the following strings: (muscle damage or creatine kinase or lactate dehydrogenase or myoglobin or soreness) and (creatine monohydrate or creatine supplementation). These free-text search strings were also used for PubMed but only for an 18-month time limit to capture publications that were still ‘in press’. For the supplementary search, screening was also conducted in Google Scholar and the reference lists of all included studies.

### Selection Process

Two experienced exercise scientists (JDC and AKR) completed the screening process. First, the abstracts (with duplicates removed) from all databases were screened using the criteria of either ‘yes’ (meeting the inclusion criteria), ‘maybe’ (possibly meeting the inclusion criteria), or ‘no’ (not meeting the inclusion criteria). Any inter-rater discrepancy was discussed with another exercise scientist (KD) until a consensus was reached. Once abstract screening was completed, the full-text articles were further screened based on the inclusion criteria.

### Data Extraction, Assessment of Quality, and Risk of Bias

The descriptive information regarding study aims, participant characteristics (e.g., age, height, body mass, body mass index [BMI], training background), research design (i.e., cross-over randomised or randomised controlled placebo), the type of biomarker for muscle damage (e.g., CK, myoglobin, and LDH) and inflammation (e.g., ILs, C-reactive protein [CRP], tumour necrosis factor [TNF]-α), the type of muscle performance measures (e.g., isokinetic/isometric knee extension), and the post-exercise time points (i.e., either 24 or 48 h post-exercise) was entered into a Microsoft Excel spreadsheet. All continuous outcome measures were extracted from each study as mean ± standard deviation (SD) to create forest plots and compare results between the CrM and placebo groups at the selected post-exercise time points. We used the PEDro rating scale as the critical appraisal tool; this originally consisted of an 11-point scoring system to assess the quality of randomised controlled trials in the Physiotherapy Evidence Database [40]. However, we modified this critical appraisal tool by incorporating four additional criteria to align the study design specifically in supplemental research and EIMD [13, 41], including (1) resistance training background of participants; (2) bioavailability of CrM; (3) reporting of active ingredients according to the manufacturer’s nutritional label; and (4) supplemental and medicinal habits of participants. As with the original PEDro criteria, the first three additional criteria were scored either as 1 (meeting the item) or 0 (not meeting the item). However, the final additional criterion was scored 2 if participants were prevented from taking CrM supplementation before the study and anti-inflammatory medication during the study, 1 if participants were prevented from taking CrM supplementation before the study or anti-inflammatory medication during the study, or 0 if the criterion was not met. Therefore, a maximum score of 16 was achievable with this modified PEDro scale, and the classification of the quality of the ratings was as follows: excellent (score 14–16); good (11–13); fair (8–10), and poor (< 7) [13]. The second author (AKR) rated each study using this modified PEDro scale. To ascertain potential publication bias of the pooled data from each study, funnel plots were created using meta-analytical software (RevMan, version 5.3, Copenhagen: The Nordic Cochrane Centre, 2014). Egger’s test was also conducted using a linear regression of the normalised effect estimates against their inverse variance to calculate the y-intercept and associated p-values [42] using a statistical software package (SPSS, v24; Chicago, IL, USA), with a *p*-value < 0.05 implicating a publication bias. However, Egger’s test was only carried out for outcome measures that consisted of at least ten studies [42] at each time point (i.e., T24 and/or T48), given that the power to detect bias for this method is low with a smaller number of studies. Participant selection bias was controlled by selecting studies of all healthy adults, irrespective of sex and training background.

### Statistical Analysis

The means and SDs of the outcome measures were extracted from each study and pooled to meta-analytically compare data between the CrM and placebo groups at 24 and 48 h following the muscle-damaging protocol. If the measure of dispersion was reported as either a 95% confidence interval or a standard error, we converted it to an SD before imputing data into the software (RevMan), based on previous recommendations [42]. Furthermore, if a study reported multiple outcome measures that measured the same phenomena (e.g., CK, myoglobin, and LDH for indirect muscle damage markers), we calculated the average to report on a singular effect estimate [43]. Once all effect estimates were combined into the statistical software, forest plots were generated using the random effects model given that the methodological design, such as participant background, muscle-damaging protocols, and biomarkers, varied between studies. Furthermore, inter-study heterogeneity was reported, with I^2^ values of 25%, 50%, and 75% interpreted as low, moderate, and high, respectively. The SMDs between the CrM and placebo groups were also derived from the forest plot at 24 and 48 h following the muscle-damaging exercise. The SMD values of 0.2, 0.5, and 0.8 were classified as small, moderate, and large, respectively [44]. The *Z*-value was also calculated to report on the effect of the pooled data between the CrM and placebo groups, with *p*-values corresponding to the level of statistical significance. We also conducted a sensitivity analysis to determine the impact of potential outliers in the forest plot.

## Results

### Systematic Literature Search

**Fig. 1:**
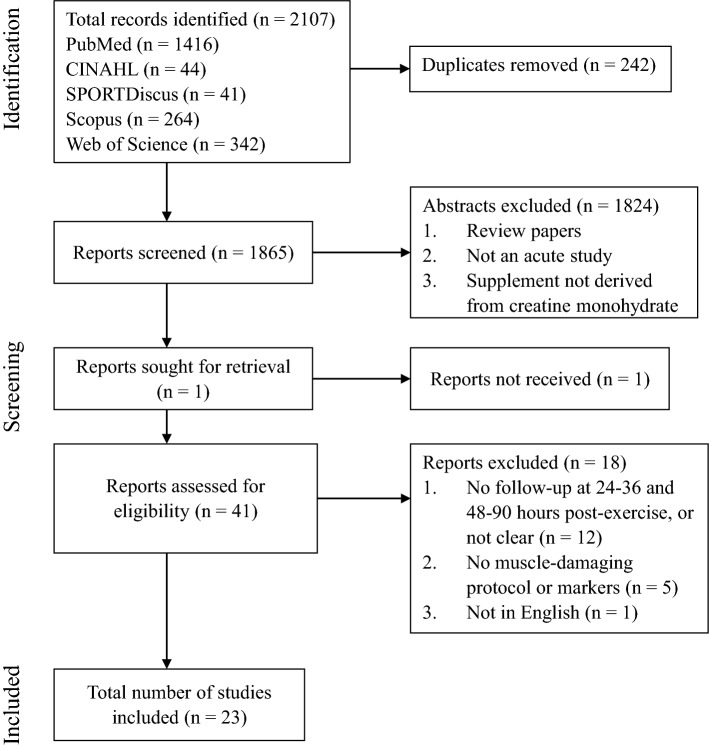
Schematic of the PRISMA flowchart

After removing 242 duplicate abstracts, 1865 abstracts from the five databases were screened according to the inclusion criteria (Fig. 1). After abstract screening, 1824 abstracts were excluded, 41 full-text articles were further screened, and the remaining 23 articles were included in this review. All studies employed a parallel design, consisting of a group that ingested CrM and a group that ingested a placebo alternative.

### Participants

From the included studies, data were extracted from 469 participants, of which 240 and 229 participants were in the CrM and placebo groups, respectively. The mean age, height, and body mass and percentage of females was 23.9 ± 10.4 years, 178 ± 5 cm, 76.9 ± 7.6 kg, and 10.4%, respectively, for the CrM group and 23.7 ± 8.5 years, 177 ± 5 cm, 77.0 ± 6.6 kg, and 10.0%, respectively, for the placebo group. As such, the physical characteristics were evenly distributed between the CrM and placebo groups (Table 1). Furthermore, there were no significant differences in the outcome measures between the CrM and placebo groups (*p* > 0.05) reported at baseline or before the muscle-damaging exercise for all but one study, suggesting that the outcome measures for both groups were relatively homogenous.

**Table 1:** Participant characteristics and baseline comparisons of outcome measures

Study	Sample size	Physical characteristics	Training background	Baseline comparisons
Atashak and Jafari [45]	CrM 9; PL 9	CrM: Age 17.41 ± 0.26 years; height 174.6 ± 4.87 cm; body mass 66.9 ± 7.89 kg PL: Age: 17.55 ± 0.35 years; height 172.08 ± 7.91 cm; body mass 70.33 ± 15.22 kg	M, young soccer players	No significant differences between conditions for all measures
Bassit et al. [46]	CrM 5; PL 6	Age 40.3 ± 2.18 years; height 178.2 ± 2.04 cm; body mass 76.3 ± 2.29 kg; triathlon participation 5.3 ± 0.80 years	M, ironman triathletes	No significant differences between conditions for all measures
Bassit et al. [47]	CrM 4; PL 4	CrM: Age 38 ± 7.1 years; height 178 ± 11.2 cm; body mass 77.4 ± 13.6 kg; triathlon participation 7.0 ± 0.7 years PL: Age 37 ± 7.7 years; height 176.5 ± 8.1 cm; body mass 76.7 ± 10.2 kg; triathlon participation 6.5 ± 2.3 years	M, ironman triathletes	No significant differences between conditions for all measures
Basta et al. [48]	CrM 10; PL 10	CrM: Age 21.6 ± 4.11 years; height 193 ± 6.02 cm; body mass 91.5 ± 7.65 kg PL: Age 21.6 ± 1.64 years; height 189 ± 5.69 cm; body mass 85.8 ± 9.21 kg	Elite rowers from senior national rowing team	No significant differences between conditions for all measures
Boychuk et al. [49]	CrM 7; PL 7	CrM: Age 24 ± 6 years; height 178.1 ± 6.2 cm; body mass: 82.4 ± 14.1 kg PL: Age 22 ± 5 years; height 181.6 ± 5.4 cm; body mass: 84.4 ± 13.1 kg	M; recreationally active	No significant differences between conditions for all measures
Brose et al. [50]	CrM 14 (8 M, 6F); PL 14 (7 M, 7F)	CrM M: Age 68.7 ± 4.8 years; height 172.0 ± 6.3 cm; body mass 84.1 ± 14.0 kg; intake 2249 ± 488 kcal/day; %PRO 16 ± 2; %CHO 54 ± 6; %Fat 29 ± 5; PRO 1.1 ± 0.3 ([g/kg]/day) CrM F: Age 70.8 ± 6.1 years; height 159.3 ± 6.9 cm; body mass 65.4 ± 16.2 kg; intake 1821 ± 457 kcal/day; %PRO 16 ± 3; %CHO 50 ± 7; %Fat 33 ± 5; PRO 1.16 ± 0.3 ([g/kg]/day) PL M: Age 68.3 ± 3.2 years; height 168.1 ± 4.9 cm; body mass 76.6 ± 9.8 kg; intake 2603 ± 587 kcal/day; %PRO 16 ± 3; %CHO 49 ± 12; %Fat 30 ± 6; PRO 1.4 ± 0.4 ([g/kg]/day) PL F: Age 69.9 ± 5.6 years; height 160.4 ± 7.7 cm; body mass 66.2 ± 14.0 kg; intake 1788 ± 507 kcal/day; %PRO 16 ± 4; %CHO 46 ± 12; %Fat 30 ± 5; PRO 1.1 ± 0.4 ([g/kg]/day)	M and F, community-dwelling older adults	Significant differences in height and intake between M and F in CrM group Significant differences in height, weight, and intake between M and F in PL group
Cooke et al. [27]	CrM 7; PL 7	CrM: Age 22.6 ± 2 years, height 173 ± 7.7 cm, body mass 77.9 ± 12 kg PL: Age 21.7 ± 3 years, height 173 ± 7.7 cm, body mass 74.4 ± 7 kg	M, untrained	No significant differences between conditions for all measures
Fernandez-Landa et al. [31]	CrM 7; PL 7	CrM: Height 183.4 ± 7.8 cm; body mass 81.2 ± 5.0 kg PL: Height 184.9 ± 2.4 cm; body mass 81.9 ± 6.3 kg	M, traditional rowers	No significant differences between conditions for all measures
Hayward et al. [34]	Protein + CrM 10; protein only 8	Age 20 ± 1 years; height 167 ± 1 cm; body mass: 63.5 ± 1.6 kg	F, healthy and non-resistance trained	No significant differences between conditions for all measures
Kaviani et al. [32]	CrM 9; PL 9	Age 23 ± 3 years; height 173 ± 6 cm; body mass 73.6 ± 5.5 kg	M, inactive	No significant differences between conditions for all measures
Machado et al. [51]	CrM 11F, 15 M; PL 11F, 12 M	CrM: Age 21 ± 2 years; height 175.0 ± 6.0 cm; body mass 64.4 ± 11.5 kg PL: Age 21 ± 2 years; height 176.1 ± 9.5 cm; body mass 64.5 ± 13.2 kg	M and F; sedentary	No significant differences between conditions for all measures
McKinnon et al. [35]	CrM 9 (5 M, 4F); PL 9 (6 M, 3F)	CrM: Age 21 ± 2 years; body mass 84.52 ± 21.02 kg PL: Age 20.78 ± 1.20; body mass 70.51 ± 14.43 kg	M and F; no upper body strength training	No significant differences between conditions for all measures
Mirzaei et al. [52]	CrM 15; PL 16	CrM: Age 19.07 ± 2.72 years; height 173 ± 5.82 cm; body mass 73.00 ± 13.09 kg PL: Age 20.00 ± 2.82 years; height 174 ± 7.38 cm; body mass 86.00 ± 16.88 kg	M, college-aged wrestlers	No significant differences between conditions for all measures
Percario et al. [33]	CrM 9; PL 9	CrM: Age 17.10 ± 1.63 years; height 182 ± 6 cm; body mass 79 ± 10 kg PL: Age 17.10 ± 1.63 years; height 181 ± 5.4 cm; body mass 80 ± 11 kg	M, handball athletes	No significant differences between conditions for all measures
Rahimi [53]	CrM 15; PL 12	CrM: Age 21.6 ± 3.6 years, height 174 ± 8 cm, body mass 71.9 ± 7.8 kg PL: Age 21.2 ± 3.2 years, height 171 ± 6 cm, body mass 69.1 ± 10.4 kg	M, resistance trained	No significant differences between conditions for all measures
Rawson et al. [36]	CrM 12; PL 11	CrM: Age 20.0 ± 0.4 years; height 176.1 ± 1.5 cm; body mass 76.6 ± 4.5 kg PL: Age 21.1 ± 1.6 years, height 179.2 ± 1.5 cm; body mass 80.7 ± 2.0 kg	M, no weight-training experience	No significant differences between conditions for all measures
Rawson et al. [38]	CrM 11; PL 11	CrM: Age 22.2 ± 1.3 years; height 176.4 ± 6.3 cm; body mass 88.4 ± 11.5 kg; training experience 6.4 ± 3.3 years PL: Age 22.1 ± 2.5 years; height 177.2 ± 5.7 cm; body mass 83.4 ± 8.2 kg; training experience 5.8 ± 3.1 years	M, resistance trained	No significant differences between conditions for all measures
Santana et al. [54]	CrM + Caf 6; PL + Caf 5	Age 20–30 years; body mass CrM + Caf 71.0 ± 15.01 kg, PL + Caf 70.8 ± 13.4 kg; running experience ≥ 6 months	M and F, healthy and active	No significant differences between conditions for all measures
Santi et al. [55]	CrM 7; PL 7	CrM: Age 18 ± 0.3 years; height 183 ± 4 cm; body mass 70.7 ± 10.7 kg PL: Age 19 ± 0.4 years; height 183 ± 6 cm; body mass 80.9 ± 6.2 kg	M; healthy and well-trained	No significant differences between conditions for all measures
Santos et al. [56]	CrM 18; PL 16	Age 25.5 ± 3.2 years; height 167.4 ± 5.4 cm; body mass 62.5 ± 7.9 kg; training experience 64. ± 1.1 years	M, trained athletes	No significant differences between conditions for all measures
Taylor et al. [37]	CrM 10; PL 9	CrM: Age 60 ± 7 years; BMI 25.8 ± 3.9 kg/m^2^ PL: Age 52 ± 6 years; BMI 28.4 ± 4.6 kg/m^2^	Did not participate in regular exercise > 2 days per week	No significant differences between conditions for all measures except between-groups age
Veggi et al. [28]	CrM 9; PL 9	CrM: Age 23.9 ± 5.5 years; height 176 ± 4.0 cm; body mass 74.2 ± 3.7 kg PL: Age 24.3 ± 4.9 years; height 178 ± 6.0 cm; body mass 74.5 ± 6.7 kg	M, healthy, not resistance trained for > 12 months	No significant differences between conditions for all measures
Wang et al. [29]	CrM 15; PL 15	CrM: Age 20 ± 2 years; height 171.93 ± 4.86 cm; body mass 67.86 ± 6.72 kg PL: Age 20 ± 1 years; height 175.93 ± 8.49 cm; body mass 70.21 ± 11.16 kg	M, athletes from football, basketball, and tchoukball team	No significant differences between conditions for all measures

*BMI* body mass index, *Caf* caffeine, *CHO* carbohydrate, *CrM* creatine monohydrate, *F* female, *M* male, *PL* placebo, *PRO* protein

### Methodological Descriptions

The most common muscle-damaging exercises were in the order of eccentric/concentric resistance exercises (11 studies), eccentric contractions (four studies), middle-to-long distance running (four studies), graded exercise test (two studies), downhill running (one study), and vertical jump test (one study) (Table 2). The most frequently reported biomarker for indirect muscle damage was CK (16 studies), followed by LDH (ten studies). Several types of inflammatory biomarkers were reported, including IL-6 (two studies), TNFα (two studies), CRP (one study), interferon-α (one study), and IL-1β (one study). Similarly, various oxidative stress markers were reported, including thiobarbituric acid reactive substances (TBARS; two studies), 8-Oxo-2′-deoxyguanosine (8-OHdG; two studies), glutamic oxaloacetic transaminase (one study), glutathione peroxidase (one study), malondialdehyde (one study), and 8-iso-prostaglandin F2α (one study). With respect to the measures of DOMS, the most common forms of visual analogue scale (VAS) consisted of 0–100 mm (four studies), followed by VAS scales of 1–10 (three studies), and 0–25 cm (one study). The most frequent muscle performance protocol consisted of isometric contractions (four studies), followed by isokinetic contraction (one study).

**Table 2:** Methodological description for route of supplement administration, muscle-damaging protocol, and the type of outcome measures

Study	Administration method	EIMD protocol	Results^a^
Atashak and Jafari [45]	One packet dissolved in 250–300 ml of water (5 g CrM plus 10 g of flavoured dextrose powder per packet equal to 0.3 g/day/kg) QID at regular intervals for 7 days	A bout of circuit weight training consisting of eight exercises, 3 sets, 15 repetitions, and 60% 1RM, with 60–90 s rest between each station	(1) CK and LDH, (2) none, (3) none, (4) none, (5) none
Bassit et al. [46]	CrM 20 g/day diluted in same amount of water divided in two equal doses for 5 days	Half-ironman competition consisting of 1.9 km swimming, 90 km cycling, and 21 km running	(1) None, (2) plasma IL-1b, IL-6, TNFα, IFNα, and PGE_2_, (3) none, (4) none, (5) none
Bassit et al. [47]	CrM 20 g/day diluted in same amount of water divided in two equal doses for 5 days	Ironman competition consisting of 3.8 km swimming, 180 km cycling, and 42 km running	(1) CK, LDH, (2) none, (3) none, (4) none, (5) none
Basta et al. [48]	CrM 20 g daily in four 5 g doses dissolved in the Isostar carbohydrate nutrient for 5 days and CrM 10 g in two 5 g doses for next 30 days	Ergometric exercise test starting with load of 50% of maximum power; the load was increased every 3 min to 60, 70, 80, and 90% of maximum power. The highest load was maintained until refusal	(1) CK, (2) TBARS, SOD, GP_X_, (3) none, (4) none, (5) none
Boychuk et al. [49]	Creatine (0.3 g/kg) in four equal aliquots (i.e., 0.075 g/kg) throughout the day, mixed in juice for 8 days	A bout of maximal eccentric contractions of 6 sets of 8 repetitions at a velocity of 90°/second, with 1 min rest between each set	(1) None, (2) none, (3) none, (4) measured using 100 mm VAS scale, (5) measured using the dominant arm for elbow flexion
Brose et al. [50]	CrM 5 g + dextrose 2 g/d mixed with juice for 14 weeks	A 14-week resistance training programme consisting of 12 exercises targeting the upper and lower body in a circuit set system. Each arm exercise was performed for 10 repetitions, and the remaining exercises were performed for 12 repetitions. Training progressed from one set of each exercise at 50% of the initial 1RM strength to three sets at 80% of 1RM over the training period. The 1RM was re-evaluated every 2 weeks and the load adjusted accordingly	(1) CK, (2) none, (3) none, (4) none, (5) measured using handgrip, ankle dorsiflexion, and knee extensor isometric strength
Cooke et al. [27]	1.5 g/kg of bodyweight/day, providing a loading dose of creatine 0.3 g/kg of bodyweight/day, mixed in water for 5 days before the exercise bout	A bout of 4 sets of 10 reps of eccentric-only contractions at 4-s cadence during leg press; leg extension and leg curl at 120% of participants' predetermined concentric 1RM, with 3 min rest between each set	(1) CK and LDH, (2) none, (3) none, (4) none, (5) measured using isokinetic/isometric knee extension
Fernandez-Landa et al. [31]	0.04 g/kg/day of CrM mixed with chocolate recovery shake for 10 weeks	10 weeks of training, consisting of six exercise sessions per week for 1.5 h/day (distributed as 60% aerobic work on a traditional boat, 30% resistance training in a gym, and 10% complementary training: injury prevention, core stability, and articular mobility)	(1) CK and LDH, (2) none, (3) none, (4) none, (5) none
Hayward et al. [34]	Micronised CrM 5 g/d with their supplemental protein for 4 weeks	4 weeks of training consisting of exercise sessions four times a week that also included a split-body workout	(1) None, (2) CRP, IL-6, (3) none, (4) measured using 100 mm VAS scale, (5) none
Kaviani et al. [32]	Creatine 0.07 g/kg/d with 250 mL grape juice, divided into two doses per day	8 weeks of resistance training consisting of three sets of 10 repetitions at 75% 1RM. Resistance increased based on a new 1RM measured every 2 weeks	(1) CK and LDH, (2) none, (3) none, (4) none, (5) none
Machado et al. [51]	20 supplemental packages containing 50% creatine and 50% dextrose at 0.6 g.kg^−1^ of bodyweight in four daily doses over 5 consecutive days	One bout of resistance training of five exercises (bench press, seated row, leg extension, leg curl, and leg press), each in three sets of ten repetitions at 75% of 1RM, with 2 min rest between each exercise	(1) CK, (2) none, (3) none, (4) none, (5) none
McKinnon et al. [35]	40 g of creatine·day^−1^ divided into two equal servings taken once in the morning and once in the evening for 5 days	One bout of 60 maximal eccentric contractions divided into 6 sets of 10 repetitions, with a 45-s rest period between repetitions on a dynamometer	(1) None, (2) none, (3) none, (4) measured using a scale ranging from 1 to 10, (5) measured using isometric elbow flexion strength
Mirzaei et al. [52]	20 g/day in four equal servings of 5 g for 7 days. The powder was mixed in 200 mL of water and ingested	Incremental exercise test to exhaustion began at 50 W for 5 min, and the power output was increased by 30 W every 3 min until voluntary exhaustion or the participant could no longer maintain a pedal cadence of 60 rpm	(1) 8-OHdG, 8-iso PGF_2α_, (2) none, (3) none, (4) none, (5) none
Percario et al. [33]	For the first 5 days, CrM was administered at a daily dose of 20 g, taken as four doses of 5 g each, dissolved in 100 ml of water. On the remaining 27 days, creatine 5 g/day, diluted in 100 ml of water was administered after training	32-day resistance training programme consisting of bench press, inclined chest fly, lat pull down, seated row, shoulder press, biceps curl, squatting, and leg extension. 3–4 sets of 3–12 repetitions and 90–180 s of rest between each set	(1) CK, urea, TBARS, TAS, uric acid, (2) none, (3) none, (4) none, (5) none
Rahimi [53]	CrM 20 g divided into four doses of 5 g for 7 days	A bout of resistance exercises consisting of 7 sets of 3–6 repetitions of bench press, leg press, lat pull down, and seated rows with 80–90% of 1RM using a pyramid loading method	(1) MDA, 8-OHdG, (2) none, (3) none, (4) none, (5) none
Rawson et al. [36]	Creatine 5 g and dextrose 7 g QID for 5 days. Participants ingested one serving of Gatorade following ingestion of the supplement at four equal intervals throughout the day	A bout of eccentric exercise test consisted of 2 sets of 25 maximal eccentric contractions, with each repetition lasting 5 s, followed by a 15-s rest between each repetition and a 5-min rest between each set	(1) CK and LDH, (2) maximum isometric force of elbow flexors, (3) none, (4) none, (5) none
Rawson et al. [38]	During the loading phase, administered creatine 0.3 g/kg of bodyweight per day in three equal doses for 5 days. Post-loading phase, creatine 0.03 g/kg of bodyweight per day for 5 days. Participants ingested the supplements with food in three equal doses per day	A bout of squat exercises consisting of 5 sets of 15–20 repetitions at 50% of 1RM with 2 min recovery between each set	(1) CK and LDH, (2) none, (3) measured using a VAS scale ranging from 1 to 10, (4) none, (5) none
Santana et al. [54]	CrM: CrM 20 g/day in four equal doses of 5 g q4h for 7 days along with 10 g of CHO. After 7 days, 5 g/day for the remaining 21 days CrHCl: Administered 6 g/day of CrHCl in four equal doses of 1.5 g q4h for 7 days along with 10 g of CHO. After 7 days, 1.5 g/day for the remaining 21 days	10 m running performance test	(1) None, (2) none, (3) measured using a VAS scale ranging from 1 to 10, (4) none, (5) none
Santi et al. [55]	Loading phase (7 days): creatine 0.3 g/kg/day associated with 1.2 g/kg/day of carbohydrate Maintenance phase (4 days): creatine 0.1 g/kg/day plus CHO supplementation, comprising maltodextrin 0.4 g/day/kg. Supplements were diluted with 300 mL of water	Vertical jump performance test	(1) CK and LDH, (2) none, (3) measured using 100 mm VAS scale, (4) none, (5) none
Santos et al. [56]	CrM 20 g diluted with the same amount of water, divided in four doses of 5 g for 5 days	30 km marathon	(1) CK, LDH, PGE_2_, TNFα, creatinine, (2) none, (3) none, (4) none, (5) none
Taylor et al. [37]	Atorvastatin 80 mg plus CrM 10 g mixed in 4–6 oz of water or juice for 4 weeks	45 min of downhill walking on a treadmill at − 15% grade and a speed set to elicit 65% of maximal heart rate	(1) CK, (2) none, (3) measured using VAS scale, (4) none, (5) none
Veggi et al. [28]	Creatine 5 g (powder form) and dextrosol 5 g QID for 6 days	A bout of four sets of barbell biceps curls to concentric failure using 75% of their predetermined 1RM, with 3 min rest between sets	(1) CK, (2) none, (3) measured using a 0–100 mm VAS scale, (4) none, (5) none
Wang et al. [29]	CrM 5 g plus dextrose 5 g dissolved in 300 mL of water QID for 6 days	4-week complex training programme consisting of six sets of 5RM half squats and plyometric jumps three times per week	(1) CK, (2) none, (3) none, (4) none, (5) none

*8-iso PGF*_*2α*_ F2-isoprostane, *8-OHdG* 8-hydroxy-2-deoxyguanosine, *CHO* carbohydrate, *CK* creatine kinase, *CrHCl* creatine hydrochloride*, CrM* creatine monohydrate*, CRP* C-reactive protein, *GPx* glutathione peroxidase, *IFN* interferon, *IL* interleukin, *LDH* lactate dehydrogenase, *MDA* malondialdehyde, *PGE*_*2*_ prostaglandin E_2,_ *q4h* every 4 h, *QID* four times daily, *RM* repetition maximum, *rpm* repetitions per minute, *SOD* superoxide dismutase, *TAS* total antioxidant status, *TBARS* thiobarbituric reactive substances, *TNF* tumour necrosis factor, *VAS* visual analogue scale, *W* watts

^a^(1) Muscle damage marker, (2) inflammatory markers, (3) oxidative stress markers, (4) delayed onset of muscle soreness, and (5) MVIC

### Methodological Quality

The scores from the PEDro scale indicated a range from fair to excellent quality (Table 3). The following PEDro items were addressed by all studies: baseline values were standardised between the CrM and placebo groups; outcome measures were reported for more than 85% of the participants; data were treated similarly irrespective of group allocation; all participants received either CrM or placebo; and appropriate statistical analyses were conducted to compare data between groups. Most studies reported the measure of dispersion (either SD, standard error, or confidence intervals); participants were randomly allocated into CrM and placebo groups; participants were instructed to refrain from pain medication/supplements and CrM supplementation before and during the study; a double-blind method was employed; and participants were homogenous. The fewest PEDro items addressed included specificity of resistance training background; concealment of allocation; and reporting of the bioavailability of the CrM supplement.

**Table 3:** PEDro ratings of all included studies

Study	1	2	3	4	5	6	7	8	9	10	11	12	13	14	15	Ratings	Quality
Atashak and Jafari [45]	1	1	1	0	1	1	0	1	1	0	1	1	1	1	1	12/16	Good
Bassit et al. [46]	1	1	1	0	1	1	1	1	1	0	1	1	1	1	1	13/16	Excellent
Bassit et al. [47]	1	1	1	0	1	1	1	1	1	0	1	1	1	1	1	13/16	Excellent
Basta et al. [48]	1	0	1	0	1	0	0	0	0	0	1	1	1	1	1	8/16	Fair
Boychuk et al. [49]	1	2	1	0	1	1	1	1	1	0	1	1	1	1	1	14/16	Good
Brose et al. [50]	1	0	1	0	0	0	0	1	1	1	1	1	1	1	1	10/16	Fair
Cooke et al. [26]	1	2	1	0	1	1	1	1	1	0	1	1	1	1	1	14/16	Good
Fernandez-Landa et al. [30]	1	1	1	1	1	1	0	1	1	0	1	1	1	1	1	13/16	Good
Hayward et al. [33]	1	1	1	0	1	1	1	0	0	0	1	1	1	1	1	11/16	Good
Kaviani et al. [31]	1	1	1	0	1	1	1	1	1	0	0	1	1	1	1	12/16	Good
Machado et al. [51]	1	1	1	1	1	0	0	1	1	0	1	1	1	1	1	12/16	Good
McKinnon et al. [34]	1	1	1	1	1	0	1	1	1	0	1	1	1	1	1	13/16	Good
Mirzaei et al. [52]	1	1	1	0	1	1	0	1	1	0	1	1	1	1	1	12/16	Good
Percario et al. [32]	1	1	1	0	1	1	0	1	1	1	1	1	1	1	1	13/16	Excellent
Rahimi [53]	1	1	1	0	1	1	1	1	1	0	0	1	1	1	1	12/16	Good
Rawson et al. [35]	1	0	1	0	1	1	1	1	1	0	1	1	1	1	1	12/16	Good
Rawson et al. [37]	1	1	1	0	1	1	1	1	1	0	1	1	1	1	1	13/16	Good
Santana et al. [54]	1	1	1	0	1	0	1	1	1	0	1	1	1	1	1	12/16	Good
Santi et al. [55]	1	1	1	0	1	1	0	1	1	1	1	1	1	1	1	13/16	Good
Santos et al. [56]	1	2	1	0	1	1	1	1	1	1	1	1	1	1	1	15/16	Excellent
Taylor et al. [36]	1	2	1	0	0	0	1	1	1	0	1	1	1	1	1	12/16	Good
Veggiv [27]	1	1	1	0	1	1	1	0	0	0	1	1	1	1	1	11/16	Good
Wang et al. [28]	1	2	1	0	1	1	0	1	1	0	1	1	1	1	1	13/16	Good

### Quantitative Analyses

**Fig. 2:**
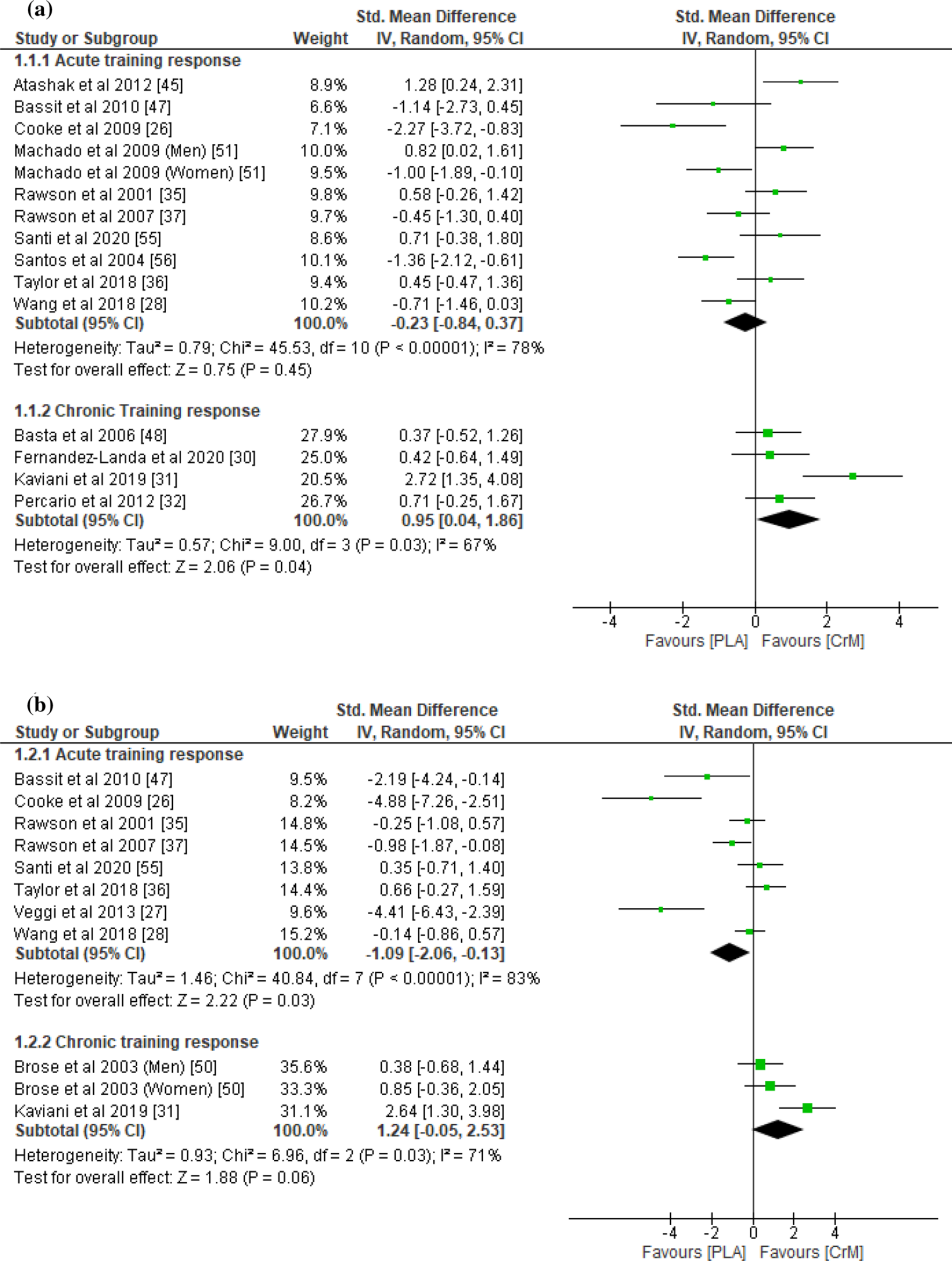
Forest plot for indirect muscle damage markers at **a** 24 and **b** 48 h after the muscle-damaging protocol. *CrM* creatine monohydrate group, *PLA* placebo group

**Fig. 3:**
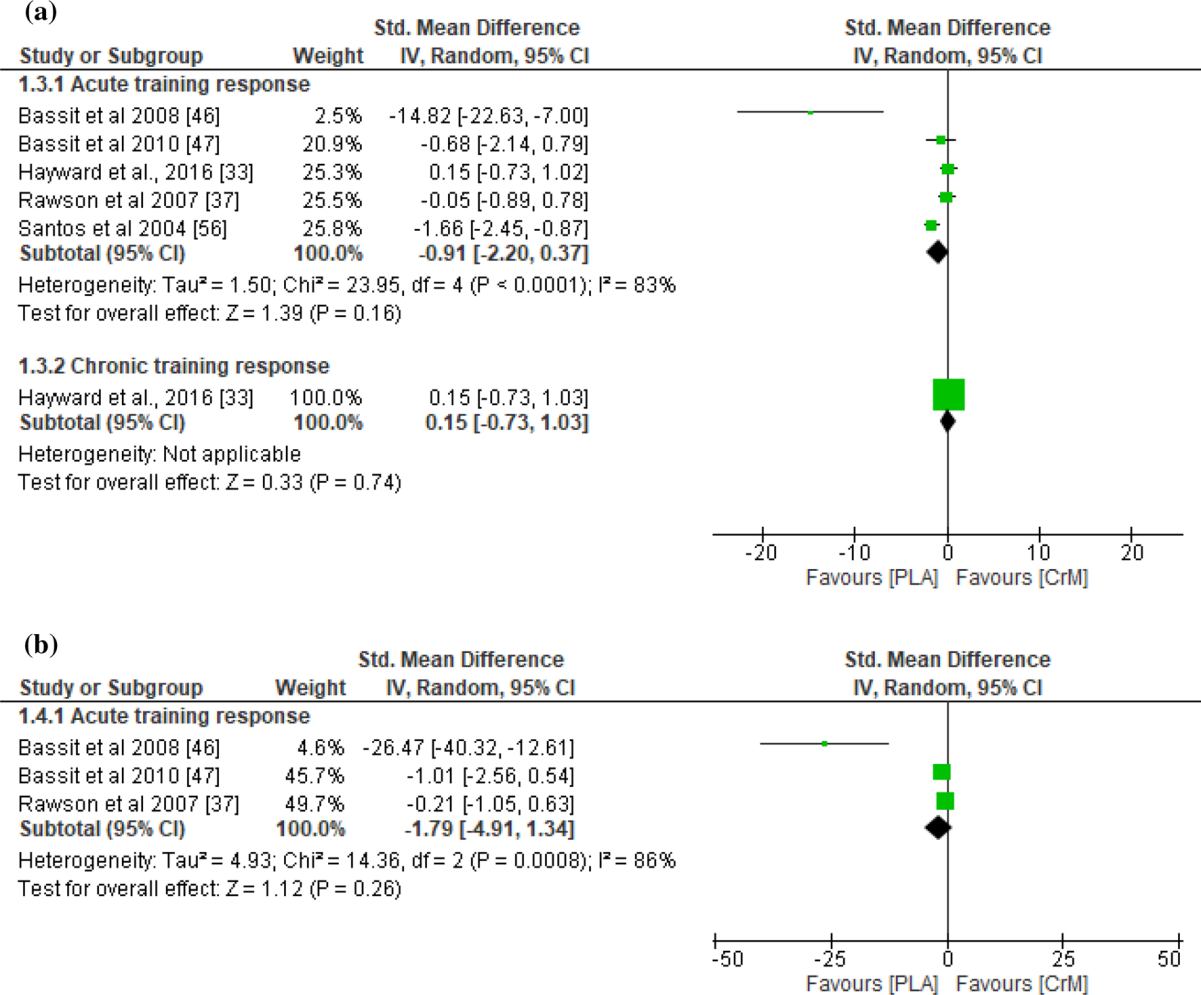
Forest plot for inflammatory markers at (**a**) 24 and (**b**) 48 h after the muscle-damaging protocol. *CrM* creatine monohydrate group, *PLA* placebo group

**Fig. 4:**
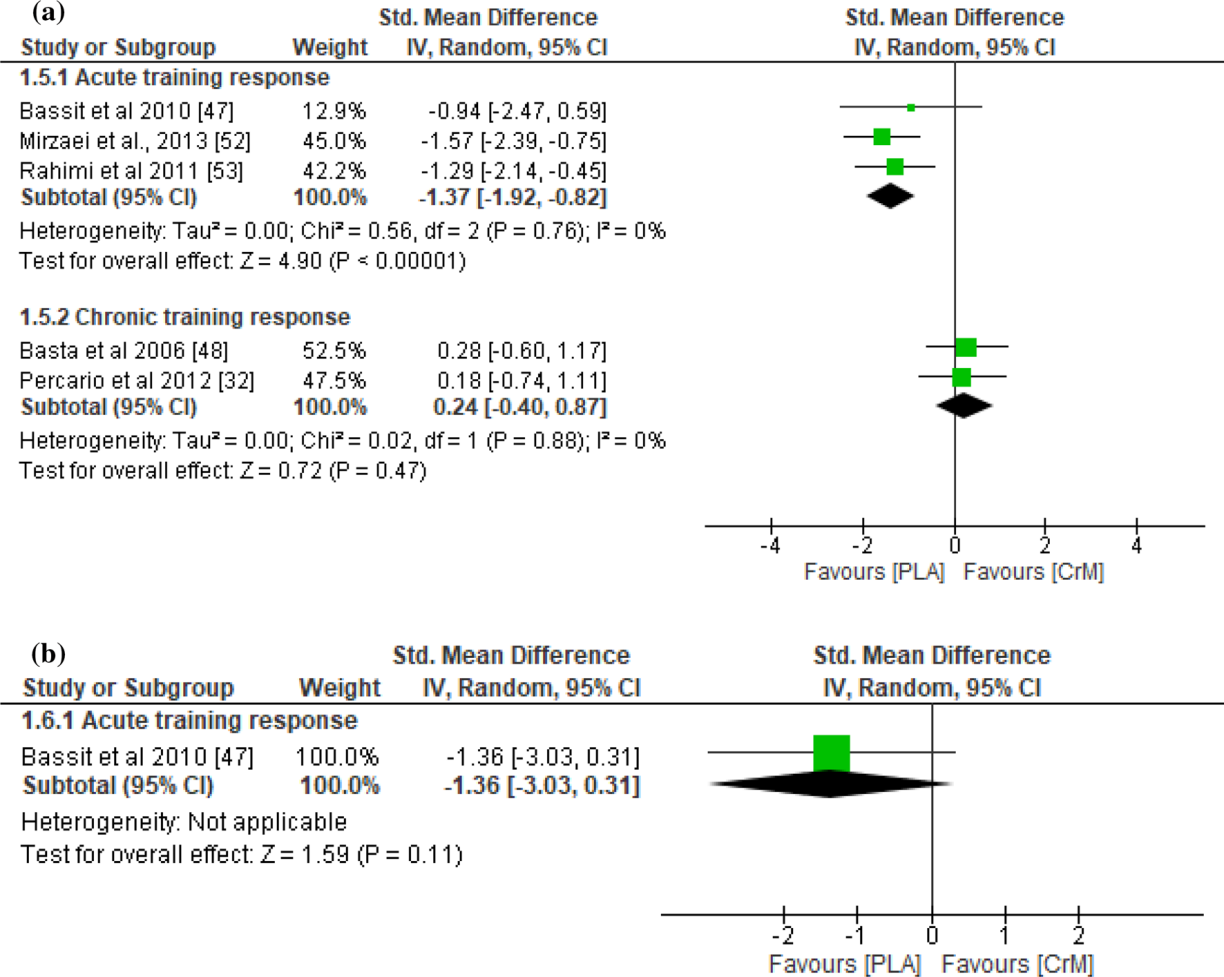
Forest plot for oxidative stress at **a** 24 and **b** 48 h after the muscle-damaging protocol. *CrM* creatine monohydrate group, *PLA* placebo group

**Fig. 5:**
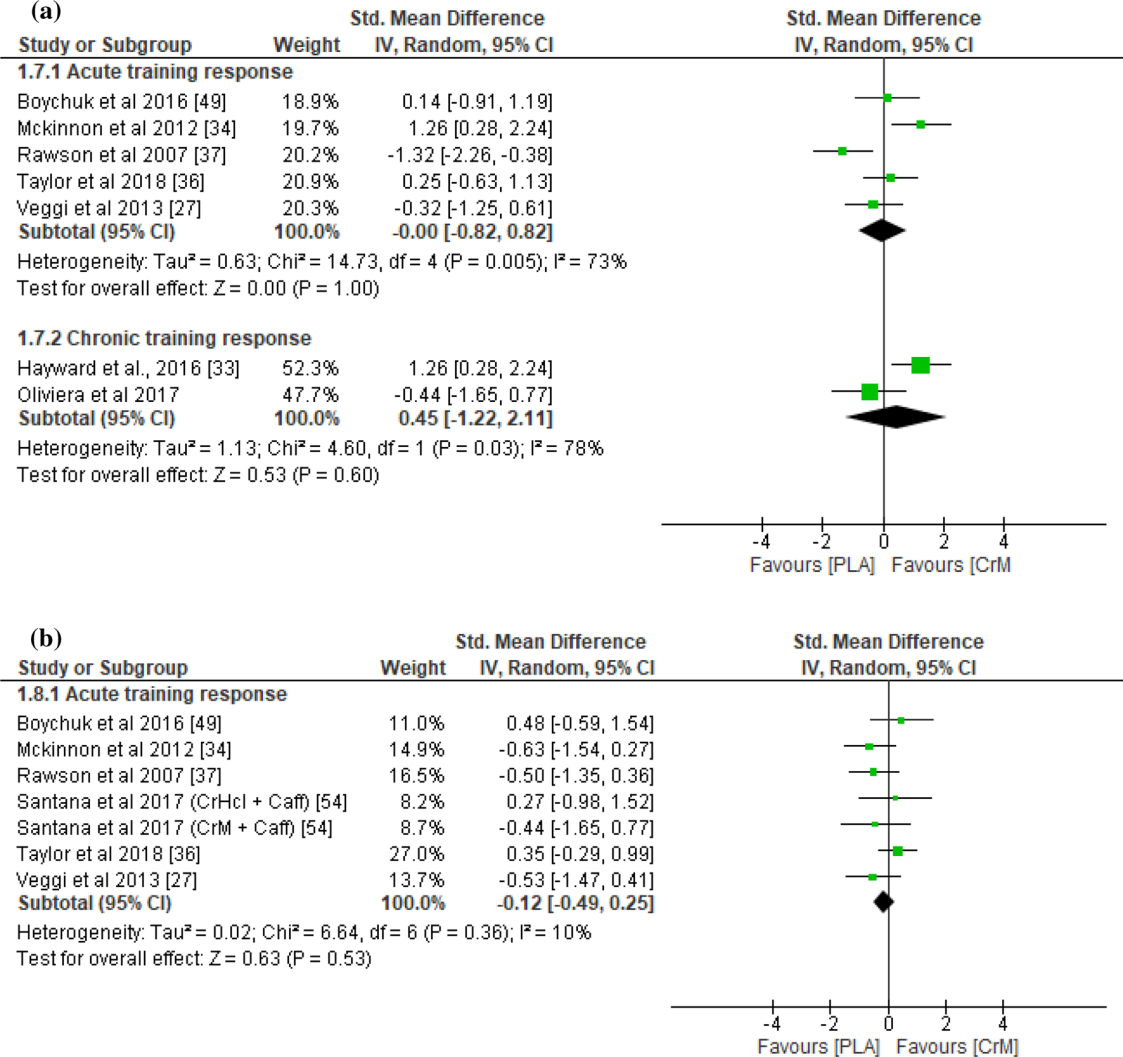
Forest plot for delayed onset of muscle soreness at **a** 24 and **b** 48 h after the muscle-damaging protocol. *CrM* creatine monohydrate group, *PLA* placebo group

**Fig. 6:**
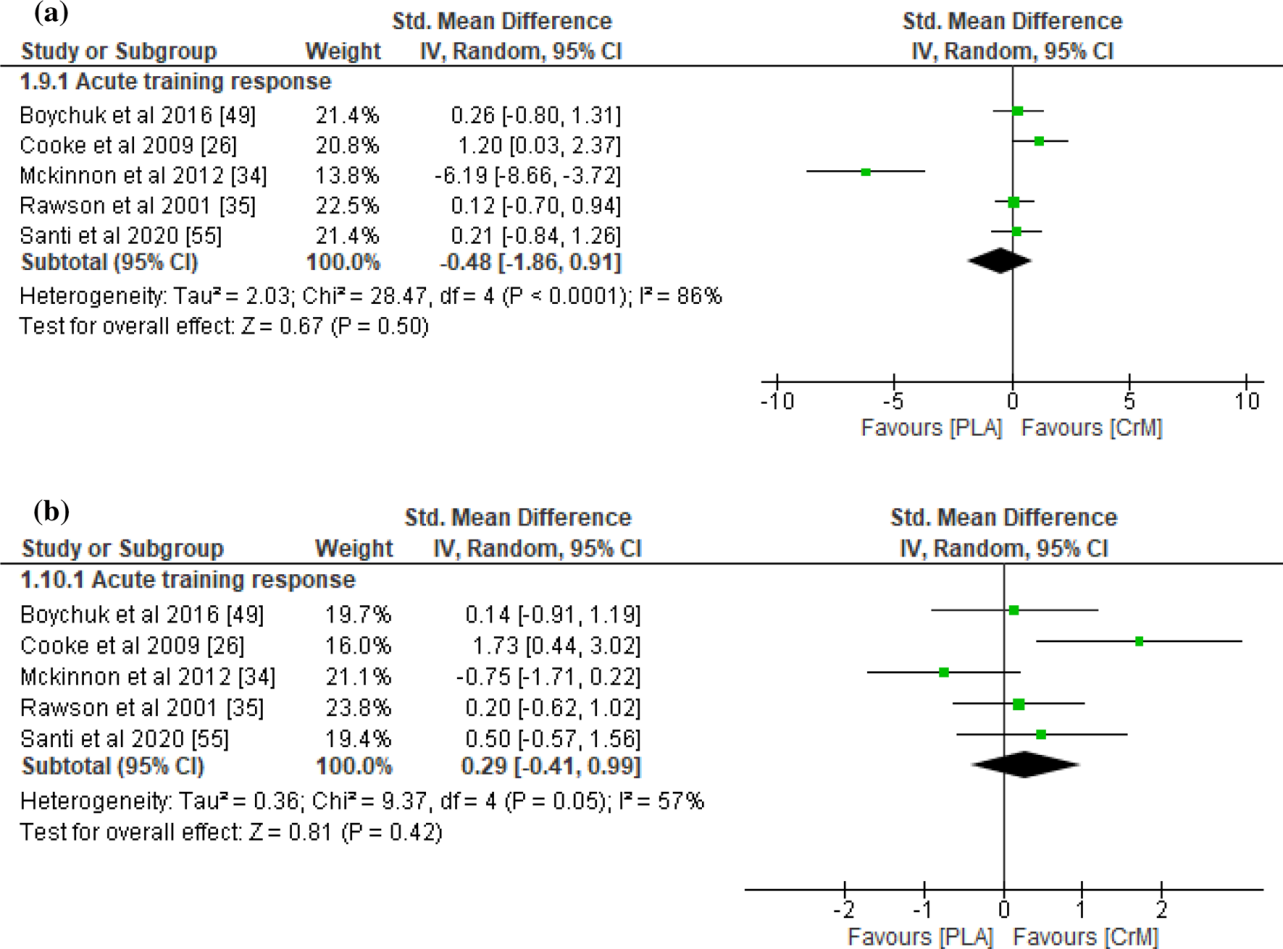
Forest plot for muscle force measures at **a** 24 and **b** 48 h after the muscle-damaging protocol. *CrM* creatine monohydrate group, *PLA* placebo group

With respect to indirect muscle damage markers (i.e., CK, LDH, and myoglobin), no significant differences were found between the CrM and placebo groups for the training response (*p* = 0.45), with small effect sizes (SMD − 0.23; *I*^2^ = 78%) at 24–36 h post-exercise (Fig. 2a). However, the indirect muscle damage markers at 48–90 h post-exercise were significantly greater in the placebo group for the acute training response (*p* = 0.03), with a large effect size (SMD − 1.09; *I*^2^ = 83%; Fig. 2b). Furthermore, indirect muscle damage markers were significantly greater in the CrM group for the chronic training response at 24 h (*p* = 0.04), with a large effect size (SMD 0.95; *I*^2^ = 67%; Fig. 2a). Although not significant (*p* = 0.06), the muscle damage markers were greater for the CrM group as a chronic training response at 48 h post-exercise, with a large effect size (SMD 1.24; *I*^2^ = 71%; Fig. 2b). The inflammatory markers appeared larger in the placebo group, with a large effect size for the acute training response at 24–36 h (SMD − 0.91; *I*^2^ = 83%; Fig. 3a) and at 48–90 h (SMD − 1.79; *I*^2^ = 86%; Fig. 3b) post-exercise, although no significant inter-group differences were evident (*p* > 0.05). Furthermore, no differences were found in inflammatory markers at 24 h post-exercise for the chronic training response (*p* = 0.74), with a small effect size (SMD 0.15). The oxidative stress markers were significantly greater in the placebo group for the acute training response at 24–36 h post-exercise (*p* < 0.001), with a large effect size (SMD − 1.37; *I*^2^ = 0%; Fig. 4a), although there were no inter-group differences for the chronic training (*p* = 0.47), with a small effect size (SMD 0.24; *I*^2^ = 0%; Fig. 4a). Although there were no inter-group differences in oxidative stress markers for the acute training response at 90 h post-exercise (*p* = 0.11), a large effect size was found with values greater for the placebo group (SMD − 1.36; Fig. 4b). There were no significant inter-group differences in DOMS for the acute and chronic training responses at 24 and 48 h (Fig. 5a and b, respectively) post-exercise (*p* > 0.05). However, the DOMS measures were greater for the placebo group, with a moderate effect size at 24 h (SMD − 0.66; *I*^2^ = 89%; Fig. 5a), but a small effect size at 48 h (SMD − 0.49; *I*^2^ = 77%; Fig. 5b) post-exercise for the acute training response. The DOMS appeared larger for the CrM group at 24 h post-exercise for the chronic training response, but with a small effect size (SMD 0.45; *I*^2^ = 78%; Fig. 5a). There were no inter-group differences in muscle force measures for the acute training responses at 24 and 48 h (Fig. 6a and b, respectively) post-exercise (*p* > 0.05), with small effect sizes (SMD − 0.48 and 0.29; *I*^2^ = 86% and 57%; respectively).

### Sensitivity Analysis

According to the sensitivity analysis, potential outliers post-exercise were identified for muscle damage at 24 h [27] and 48 h [28], inflammatory markers at 24 h [46] and 48 h [46], oxidative stress at 24 h [47], DOMS at 24 h [38] and 48 h [28], and muscle force at 24 h [35] and 48 h [36]. When excluding these outliers, neither heterogeneity nor effect estimates were influenced for post-exercise muscle damage at 24 h (*I*^2^ = 76%; SMD − 0.08; *p* = 0.45), oxidative stress at 24 h (*I*^2^ = 0%; SMD − 1.44; *p* < 0.001), DOMS at 48 h (*I*^2^ = 14%; SMD − 0.06; *p* = 0.78), and muscle force at 48 h (*I*^2^ = 68%; SMD 0.34; *p* = 0.48). However, post-exercise muscle damage at 48 h changed from large to moderate for SMD (I^2^ = 76%; SMD − 0.65; p = 0.14), inflammatory markers at 24 h changed from large to moderate for heterogeneity and from large to moderate for SMD (*I*^2^ = 73%; SMD − 0.56; *p* = 0.23), inflammatory markers at 48 h changed from large to low for heterogeneity and from large to small for SMD (*I*^2^ = 0%; SMD − 0.39; *p* = 0.30), DOMS at 24 h changed from moderate to low for heterogeneity (*I*^2^ = 45%; SMD 0.33; *p* = 0.32), and muscle force at 24 h changed from large to low for heterogeneity (*I*^2^ = 0%; SMD 0.36; *p* = 0.15).

### Risk of Bias

On visual inspection, the funnel plots appeared relatively symmetrical and evenly distributed for muscle damage at 24 h post-exercise (electronic supplementary material [ESM]-1a), DOMS at 24 and 48 h post-exercise (ESM-2a and 2b, respectively), oxidative stress at 24 h post-exercise (ESM-3a), and muscle force measures at 24 and 48 h post-exercise (ESM 4a and 4b). However, the studies appeared to congregate towards the top of the funnel plot for muscle damage at 48 h post-exercise (ESM-1b), inflammatory markers at 24 and 48 h post-exercise (ESM 5a and 5b, respectively), and oxidative stress at 48 h post-exercise (ESM-3b). Egger’s test did not indicate publication bias for the muscle damage markers at 24 h (*p* = 0.55) and 48 h post-exercise (*p* = 0.17), although we did not conduct this test for the other outcome measures because the number of studies was insufficient.

## Discussion

This systematic review examined the effects of CrM supplements to reduce the signs and symptoms of EIMD. According to the meta-analysis, indirect markers of muscle damage and inflammatory and oxidative stress markers were lower in the CrM group after the muscle-damaging exercise as an acute training response, with large effect size calculations. Conversely, indirect muscle damage markers were higher in the CrM group as a chronic training response, also with large effect size calculations. Although no inter-group differences were identified for DOMS measures, the values appeared lower for the CrM group, with moderate effect size calculations, as an acute training response. There were no inter-group differences in muscle performance measures, with small effect size calculations for both acute and chronic training responses. Overall, there was some evidence that CrM reduced the level of EIMD as an acute training response, but exacerbated it for indirect muscle damage and inflammatory markers as a chronic training response, although this was dependent on the period of EIMD (i.e., 24–36-h or 48–90-h post-exercise). Furthermore, CrM did not appear to aid in the recovery of muscle performance measures following muscle-damaging exercises.

The findings from our meta-analysis were in line with the meta-analysis by Northeast and Clifford [24], whereby indirect muscle damage markers were only significantly lower for CrM at 48 h post-exercise, with minimal differences observed for DOMS and muscle force measures. Interestingly, muscle force measures showed a large effect size (SMD − 0.86) in the meta-analysis by Northeast and Clifford [24] at 24 h post-exercise, whereas our meta-analysis only showed a small effect size (SMD − 0.48) in the same direction. The discrepancy in data analysis may be due to the inclusion of the recently published study by Santi et al. [55], who reported a small effect size (SMD 0.21), but which was not included in the meta-analysis by Northeast and Clifford [24]. Furthermore, comparing our data on the oxidative stress and inflammatory markers to previous work is currently difficult because the current meta-analysis and systematic review is the first to examine the effects of CrM on these markers during periods of EIMD. Nonetheless, previous work has employed the same approach, by conducting meta-analysis to examine the ergogenic aids of supplements during periods of EIMD, but with plant extracts. Doma et al. [13] recently conducted a systematic review and meta-analysis of fruit-derived supplements on EIMD measures, showing significantly lower values for inflammation and oxidative stress with fruit-derived supplements than with placebo 24–48 h after the muscle-damaging exercises. However, their SMD values only ranged from 0.20 to 0.34, which is substantially smaller than the SMD values of inflammation and oxidative stress identified in the current meta-analyses for CrM as an acute training response (SMD 0.91–1.79). In another recent meta-analysis that examined the effect of root plant supplements on EIMD measures [15], inflammation was significantly lower than with placebo conditions at 24–48 h post-exercise. However, their SMD values ranged from 0.09 to 0.34, which again, are notably less than the SMDs of the current meta-analyses. Thus, CrM appears to provide greater protection against inflammation and oxidative stress than supplements derived from plants as an acute training response. These differences between meta-analyses may be attributed to the distinct biochemical constituents of CrM and plant-based extracts.

Several mechanisms have been proposed to explain how CrM ameliorates the signs and symptoms of EIMD, although they are not completely clear. First, a plethora of evidence demonstrates that muscle-damaging exercises increase inflammation, and this inflammatory response is believed to augment markers of EIMD via the secondary muscle damage response [1]. Furthermore, the elevation in inflammatory response also generates reactive oxygen species, which increases oxidative stress [57]. These processes cause further damage to already damaged and non-damaged muscle fibres, which accelerates myocyte membrane damage via peroxidation [58]. However, supplementation of CrM is believed to counteract increases in both inflammation and oxidative stress, which would limit further damage to skeletal muscle [59]. Our meta-analysis partly confirms this hypothesis, whereby the CrM group exhibited lower inflammatory and oxidative stress markers than the placebo group for up to 48 h post-exercise, with large effect sizes. However, we were unable to analyse the anti-inflammatory and antioxidant capacities of CrM because an insufficient number of studies examined these markers. Deminice and Jordao [25] showed that CrM supplementation decreased TBARS and increased total antioxidant capacity; however, this study was conducted in rats so further research is necessary to confirm the anti-inflammatory and antioxidant roles and associated mechanisms of CrM in humans.

The current meta-analysis did not identify significant inter-group differences in DOMS between the CrM and placebo groups. One reason for this trend may be the subjectivity and limited inter-day reliability of the instruments used to measure DOMS [60], which would require a greater sample size to identify significant differences. Nonetheless, the values appeared smaller for the CrM group than for the placebo group, with a moderate effect size for up to 24 h post-exercise. It has been suggested that the mechanical damage of the intermediate myofilaments activates group III and IV afferent nociceptors, resulting in symptoms of DOMS [2]. In the current systematic review, supplementation of CrM exhibited lower levels of indirect muscle damage, inflammation, and oxidative stress markers. Thus, we can assume that the antioxidant and anti-inflammatory capabilities of CrM reduced the activation of nociceptors, thereby minimising the symptoms of DOMS following muscle-damaging exercises.

Impaired muscle performance is a common occurrence during periods of EIMD. Possible explanations include alterations in the length of sarcomere caused by mechanical damage of muscle fibres, impaired excitation–contraction coupling, and influx of calcium concentrations, leading to prolonged deficits of muscular contractility [1]. However, the increase in intra-muscular phosphocreatine following the ingestion of CrM accelerates re-phosphorylation of adenosine triphosphate. This process sustains sarcoplasmic reticular calcium pump function by decreasing cytosolic calcium concentration [61], which is believed to enhance recovery of muscular function following the ingestion of CrM. Interestingly, the current meta-analysis showed no inter-group differences between the CrM and placebo groups for muscle performance measures, with small effect size calculations. The limited effect of CrM on muscle performance could be attributed to the variety of methods used to measure muscle force (e.g., vertical jump height vs. isometric contractions) and the muscle groups assessed (e.g., knee extensors vs. elbow flexors). This results in a complex interaction of a number of different biomechanical and physiological factors influencing performance. Further, the limited number of studies assessing this specific outcome measure could be another reason for the absence of significant results. In fact, Doma et al. [13] also suggested similar confounders when they reported the lack of any differences in muscle performance measures with root plant supplements during periods of EIMD in their meta-analysis. Thus, more research is necessary to confirm the effects of CrM as a supplement to benefit the recovery of muscle strength. In this regard, the use of valid neuromuscular measures in low-complex tasks would be recommended to better isolate the effects of CrM on neuromuscular function.

Although the current meta-analysis showed that CrM may minimise the level of EIMD following muscle-damaging exercises as an acute training response (i.e., one bout of muscle-damaging exercises), greater levels of EIMD were found as a chronic training response (i.e., the last bout of muscle-damaging exercises from several weeks of training in conjunction with supplementation of CrM). Furthermore, this reversed trend was observed by all studies included in this systematic review that examined chronic training responses, which strengthens the possibility that CrM could also exacerbate the level of EIMD depending on the method of delivery. This paradoxical effect was unexpected, given that studies typically implement CrM as a supplement to reduce markers of EIMD. However, the majority of authors of studies that examined the chronic training responses suspected that CrM might have augmented the level of EIMD to a greater extent than placebo because of enhanced training adaptations. For example, Kaviani et al. [32] suggested that the participants in their CrM group exhibited significantly greater CK measures than the placebo group after 8 weeks of resistance training with supplements because of an accelerated progression of resistance training intensity in the CrM group. Furthermore, the increase in intra-muscular phosphocreatine stores may have allowed for a higher training volume with the CrM group, resulting in greater damage to the muscles in a dose–response manner. Similar trends were also observed in the study by Brose et al. [50], who speculated that long-term CrM supplementation with several weeks of resistance training would increase total muscle creatine and fat-free mass, thereby augmenting the concentration of creatinine in the plasma. Thus, although CrM may provide protection against muscle damage in the short term following the first few training sessions, this trend may be reversed with longer-term supplementation and training. Possible strategies to ameliorate greater levels of EIMD as a chronic training response may be to consider a combination of oral supplements to manage EIMD, such as combining CrM with other supplements (e.g., herbal supplements, fruits, branched chain amino acids). Nonetheless, it is important to note that this heightened level of physiological stress may be necessary for enhanced adaptations to occur given that the CrM groups also exhibited greater training adaptations.

A number of issues need to be addressed in future research. First, more studies should consider assessing the bioavailability of CrM: > 70% of studies included in this systematic review did not consider this factor. This should be an essential component of these studies, given that the ergogenic effects of CrM for recovery reflect the absorption rate of CrM, which in turn, is the most effective method of confirming the placebo effect. Second, studies reported only certain biomarkers to gain insight into the mechanisms contributing to the protective effects of CrM on the signs and symptoms of EIMD. Future studies should incorporate a range of biomarkers to develop a better understanding of muscle damage (including collagenase matrix metalloproteinase and B-cell lymphoma 2-associated athanogene 3) [62], anti-inflammatory and antioxidant effects of CrM during EIMD and proteostasis, and the potential ergogenic role of CrM for muscle recovery. Finally, although some of the studies included in this systematic review combined both males and females in their sample, whether sex affects the ergogenic effects of CrM during periods of EIMD remains unclear, warranting further research.

A number of limitations in this systematic review should be identified. First, several types of muscle-damaging protocols and participant characteristics were amalgamated meta-analytically, which may have impacted the degree of change in outcome measures. This is an important consideration because the level of EIMD is dependent on the training background [63], mode [64], and intensity [65] of exercise. Second, the rate of recovery with CrM supplementation appeared to vary between 24–36 h and 48–90 h post-exercise for each outcome measure, making precise recommendations difficult for each outcome measure. Third, the dosage of CrM was distinct between studies, also causing difficulty in providing exact recommendations on the amount of CrM required to optimise recovery following strenuous exercise. Thus, more research is necessary to improve recommendations on the dosage method for CrM supplementation and the time course recovery following strenuous exercises. Fourth, markers of muscle damage and oxidative stress may increase more than 90 h post-exercise in some cases [66], limiting the possibility of capturing a precise trend over time after strenuous exercises. Finally, we excluded all studies published in languages other than English, which may have introduced cultural bias.

## Conclusion

Our systematic review and meta-analysis demonstrated a paradoxical effect of CrM supplementation, where the level of EIMD was reduced for several days after muscle-damaging exercises as an acute training response, but this trend was reversed as a chronic training response. Accordingly, coaches and athletes could consider incorporating CrM to aid in the acute recovery of strenuous training sessions, with the expectation that training-induced physiological stress and EIMD symptoms may be augmented following long-term use of CrM. However, further research is necessary to determine the ergogenic effects of CrM as a recovery supplement for muscular contractility during periods of EIMD.

## Supplementary Information

Below is the link to the electronic supplementary material.

Supplementary file1 (PPTX 51 kb)

Supplementary file2 (PPTX 49 kb)

Supplementary file3 (PPTX 48 kb)

Supplementary file4 (PPTX 49 kb)

Supplementary file5 (PPTX 50 kb)

## Declarations

### Availability of data and material

Data will be provided upon request.
