# Supplementary figures and images for: The Paradoxical Effect of Creatine Monohydrate on Muscle Damage Markers: A Systematic Review and Meta-Analysis
Source: Sports Med. 2022 Feb 26;52(7):1623–45. doi: 10.1007/s40279-022-01640-z (PMC9213373; doi:10.1007/s40279-022-01640-z)

## Slide 1
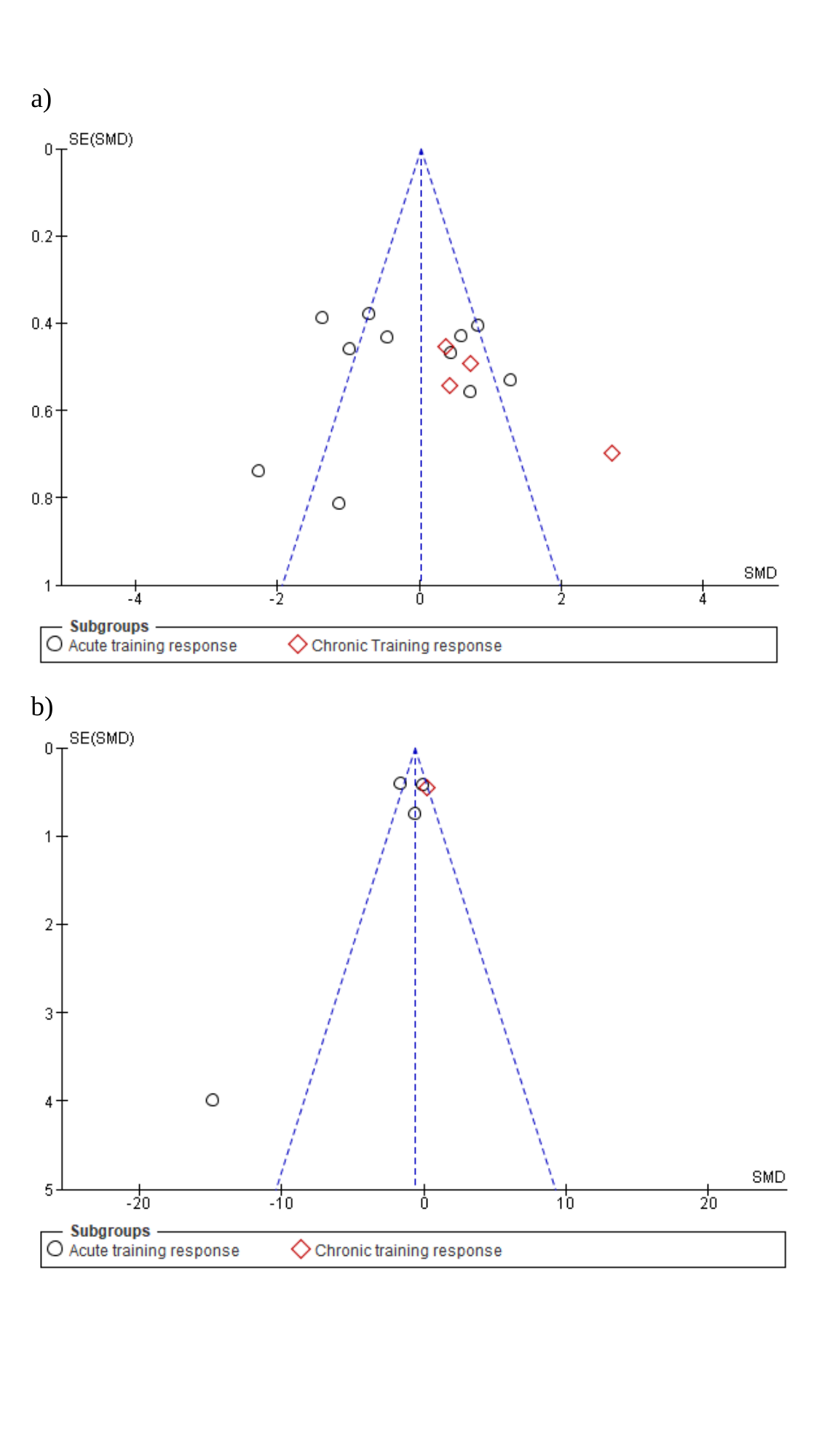

a)
b)

Supplement: Supplementary file 1 — Supplementary file1 (PPTX 51 kb) [file 40279_2022_1640_MOESM1_ESM.pptx]

## Slide 1
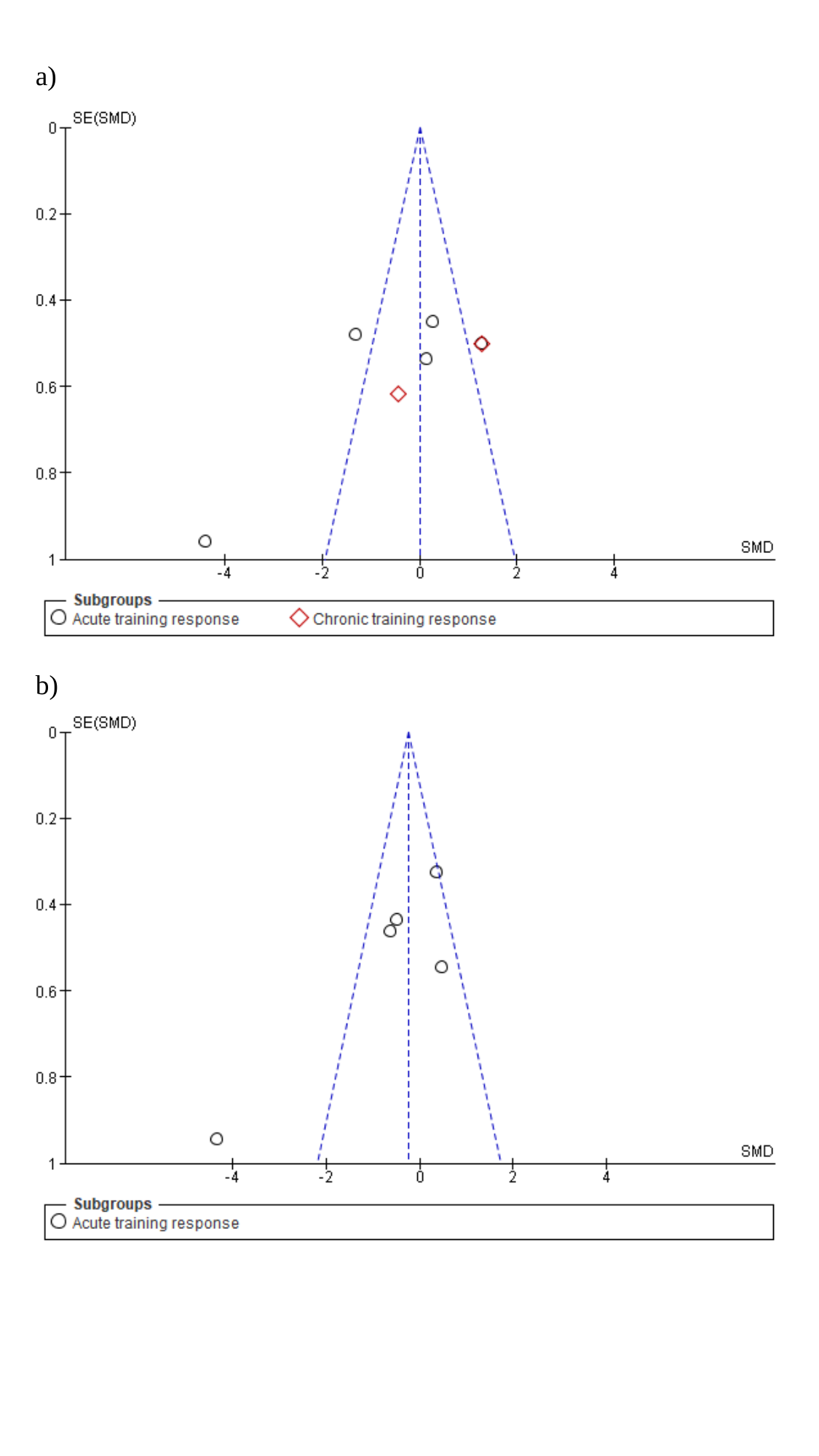

a)
b)

Supplement: Supplementary file 2 — Supplementary file2 (PPTX 49 kb) [file 40279_2022_1640_MOESM2_ESM.pptx]

## Slide 1
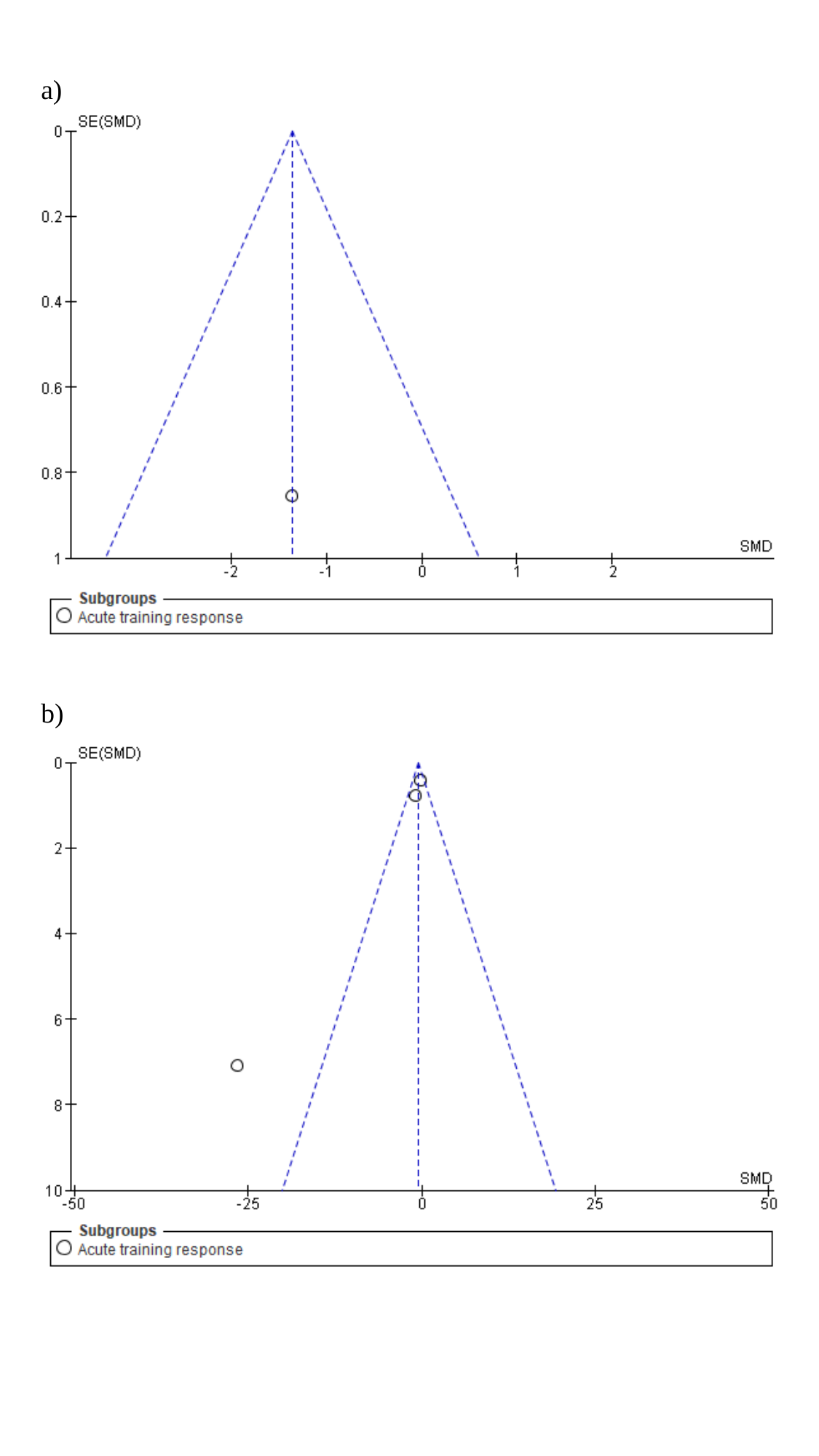

a)
b)

Supplement: Supplementary file 3 — Supplementary file3 (PPTX 48 kb) [file 40279_2022_1640_MOESM3_ESM.pptx]

## Slide 1
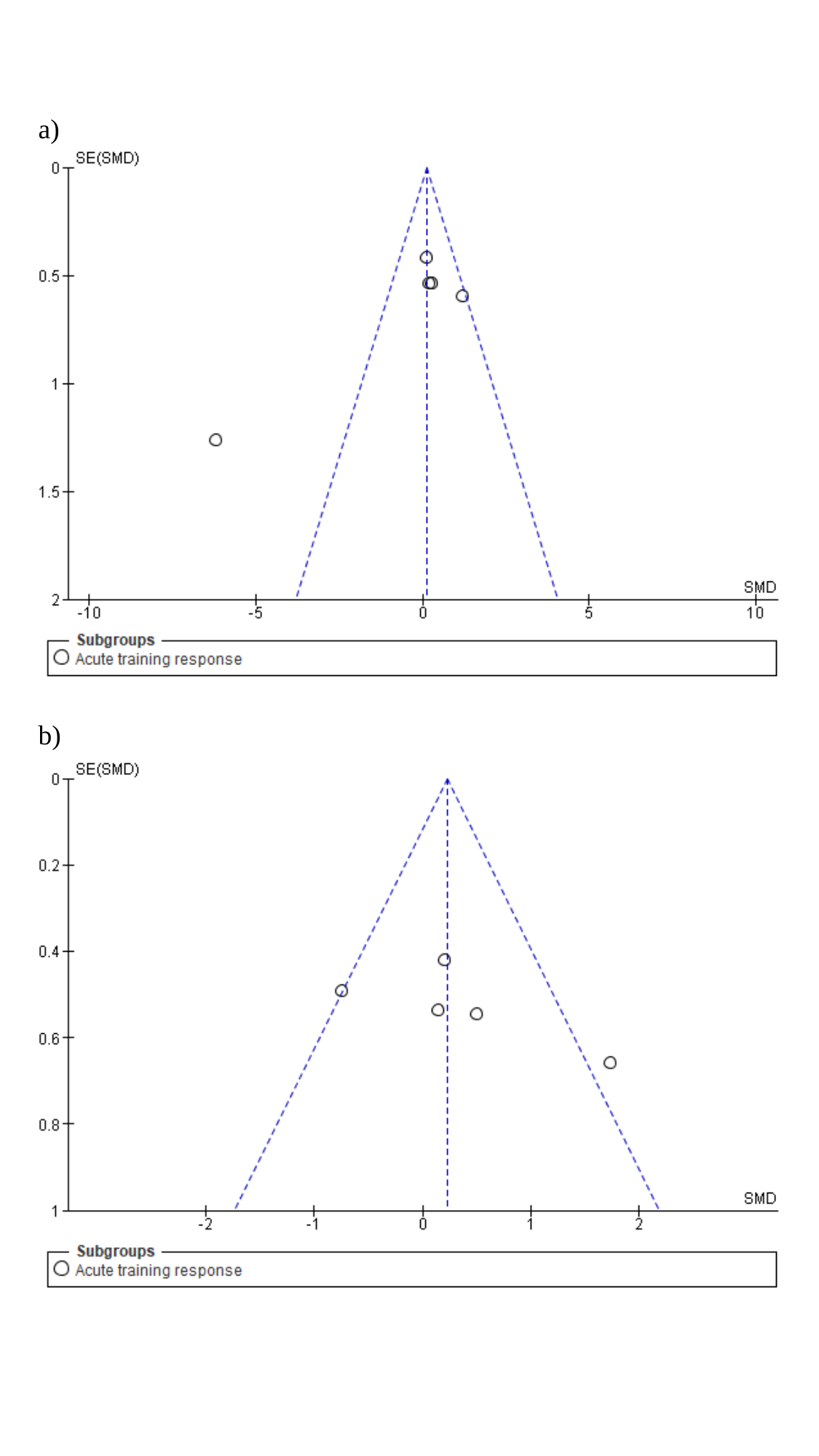

a)
b)

Supplement: Supplementary file 4 — Supplementary file4 (PPTX 49 kb) [file 40279_2022_1640_MOESM4_ESM.pptx]

## Slide 1
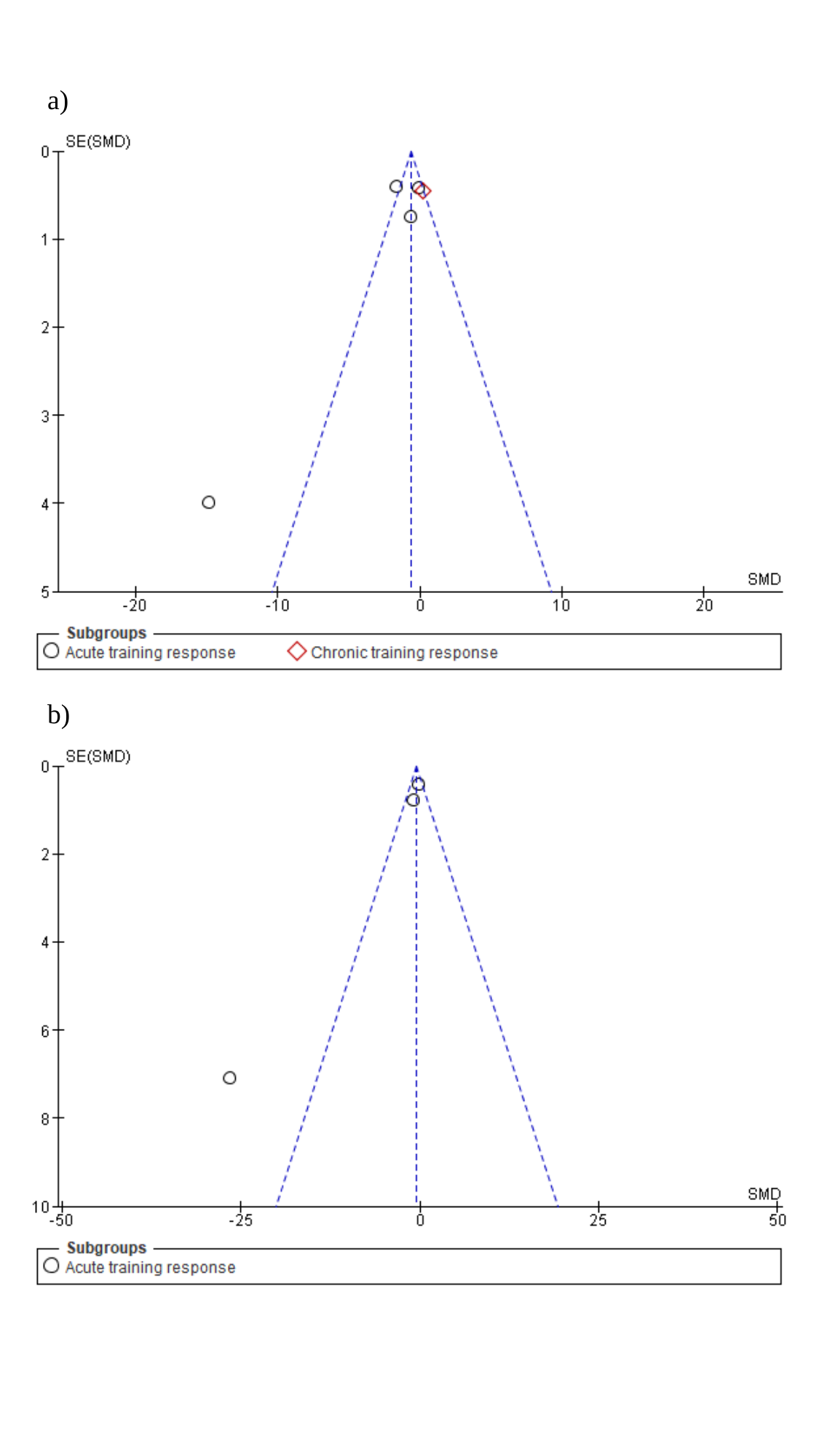

a)
b)

Supplement: Supplementary file 5 — Supplementary file5 (PPTX 50 kb) [file 40279_2022_1640_MOESM5_ESM.pptx]
